# Older Adults Seek Out Congregate Nutrition Programs for Socialization

**DOI:** 10.1093/geroni/igaa057.060

**Published:** 2020-12-16

**Authors:** Kristen Robinson, Susan Jenkins

**Affiliations:** 1 Social & Scientific Systems, Inc., Frederick, Maryland, United States; 2 Administration for Community Living, Washington, District of Columbia, United States

## Abstract

Social connectedness is vital for healthy aging. Older adults often have fewer opportunities to socialize due to reasons such as illness, death of spouse, and mobility limitations. The Older Americans Act Congregate Nutrition Program provides meals and related nutrition services in group settings to people age 60 and over. Until now, the contribution of congregate meals to the socialization of older adults has been primarily anecdotal. However, according to data from the 2019 National Survey of Older Americans Act Participants, over 40% of the 1.5 million people receiving congregate meals said that they started attending to socialize with other people. According to the data, two-thirds report their social opportunities have increased since becoming involved with these services. To measure the impact of program participation on socialization outcomes, an evaluation compared congregate program participants to non-participants. Findings from regression-adjusted socialization outcomes found that congregate meal participants were less likely to screen positively for depression (18% vs. 24% p<.05) and have greater satisfaction with their socialization opportunities (94% vs. 86% p<.01), yet there was no significant difference in a measure of perceived loneliness. Based on these results, we used weighted, bivariate tests to detect differences between congregate meal participants who were satisfied with the socialization opportunities to those who were not. Our findings suggest a significant relationship between number of days per week participants attended congregate meals and satisfaction with socialization, X2 (2, N = 1,072) = 7.5, p = < .05.

